# Coinfections and antimicrobial use in patients hospitalized with coronavirus disease 2019 (COVID-19) across a single healthcare system in New York City: A retrospective cohort study

**DOI:** 10.1017/ash.2022.51

**Published:** 2022-05-10

**Authors:** Prithiv J. Prasad, Jordan Poles, Ioannis M. Zacharioudakis, Yanina Dubrovskaya, Dinuli Delpachitra, Eduardo Iturrate, Sigridh Muñoz-Gómez

**Affiliations:** 1 NYU Grossman School of Medicine, New York City, New York; 2 NYU Long Island School of Medicine, Mineola, New York

## Abstract

**Background and objective:**

With the coronavirus disease 2019 (COVID-19) pandemic, rates of in-hospital antimicrobial use increased due to perceived bacterial and fungal coinfections along with COVID-19. We describe the incidence of these coinfections and antimicrobial use in patients hospitalized with COVID-19 to help guide effective antimicrobial use in this population.

**Setting:**

This study was conducted in 3 tertiary-care referral university teaching hospitals in New York City.

**Methods:**

This multicenter retrospective observational cohort study involved all patients admitted with COVID-19 from January 1, 2020, to February 1, 2021. Variables of interest were extracted from a de-identified data set of all COVID-19 infections across the health system. Population statistics are presented as median with interquartile range (IQR) or proportions with 95% confidence intervals (CIs) as indicated.

**Results:**

Among 7,209 of patients admitted with COVID-19, 663 (9.2%) had a positive culture from the respiratory tract or blood sometime during their initial hospital admission. Positive respiratory cultures occurred found in 449 (6.2%) patients, and 20% were collected within 48 hours of admission. Blood culture positivity occurred in 334 patients (4.6%), with 33.5% identified within 48 hours of admission. A higher proportion of patients received antimicrobials in the first wave than in the later pandemic period (82.4% vs 52.0%). Antimicrobials were prescribed to 70.1% of inpatients, with a median of 6 antimicrobial days per patient. Infection-free survival decreased over the course of hospitalization.

**Conclusions:**

We detected a very low incidence of coinfection with COVID-19 at admission. A longer duration of hospitalization was associated with an increased risk of coinfection. Antimicrobial use far exceeded the true incidence and detection of coinfections in these patients.

Healthcare systems have struggled to cope with the surge in coronavirus disease 2019 (COVID-19) cases caused by severe acute respiratory coronavirus virus 2 (SARS-CoV-2), leading to calls for optimizing healthcare resource utilization.^1,2^ New York City was an early epicenter of the pandemic, reaching a peak of 6,578 cases diagnosed on April 6, 2020, alone.^3^ With an alarmingly high mortality rate among patients with severe disease,^4^ expensive therapeutics, and stressed healthcare systems, optimal treatment of comorbid conditions and allocation of already scarce resources remains a priority in current daily practice.^5^ Widespread use of antimicrobials to treat suspected bacterial coinfections during the pandemic threatens to derail efforts that combat another looming threat to global health security—the emergence of antimicrobial resistance.^6–9^

Study of previous influenza pandemics has emphasized the importance of bacterial coinfections caused by common oropharyngeal colonizers in patients’ outcomes, and autopsy studies suggest that such coinfections complicated nearly all influenza deaths in the 1918 influenza pandemic and up to 50% of 2009 influenza A (H1N1) pandemic, or between 18% and 34% using contemporary testing.^10^ Additionally, antibiotic resistance associated with Influenza activity and antibiotic overuse is increasing.^11^ In one study, 41% of patients with acute respiratory infections during influenza season received antibiotics in the absence of bacterial coinfections.^12^

It is unclear whether coinfection with other respiratory pathogens worsens outcomes in patients with COVID-19 infection. Early reports have raised concerns that bacterial and fungal coinfections play a role in the morbidity and mortality of patients hospitalized with COVID-19. The rates of coinfection among studies of patients with COVID-19 disease range widely from 0.6% in China to 20.7% in California^13,14^; a meta-analysis showed a 7% overall incidence of bacterial coinfection.^15^

Here, we have described the incidence of coinfections in patients admitted with COVID-19 in a multihospital single healthcare system in New York, and we have quantified the antimicrobial use in this population to help guide antimicrobial treatment and stewardship.

## Methods

### Study design, setting, and participants

We conducted a retrospective observational cohort study among the 3 teaching hospitals that comprise the NYU Langone Health System in New York City (NYULH), namely NYU Brooklyn, NYU Long Island, and Tisch hospital. Using data collected between January 1, 2020, and February 1, 2021, we evaluated all initial inpatient admissions of patients over 18 years of age with a positive SARS-CoV-2 nasopharyngeal polymerase chain reaction (PCR) test documented within 14 days before or after the day of admission. The study period was divided into the first wave and later pandemic periods, based on the nadir of the epidemic curve for inpatient admissions to the hospital. This study was conducted when as healthcare capacity in New York City was strained during the first wave, which potentially contributed to a higher risk of secondary infection. Patients were followed for 28 days from the date of admission to our hospital system or until discharge, transfer out of the hospital, or death, whichever came first, at which point data were censored. Readmissions and patients who were transferred from outside hospitals were excluded.

### Outcome measures and follow-up

The primary study outcome was the incidence of bacterial, fungal, and viral coinfections within 48 hours of admission among patients hospitalized with COVID-19. Coinfection was defined as the growth of a microorganism in bacterial or fungal clinical cultures or detection of a virus on a validated upper respiratory PCR-based diagnostic test <48 hours from the time of admission. Secondary outcomes were the incidence of secondary infections among patients hospitalized with COVID-19 infection, infection-free survival in hospitalized patients, and rates of antimicrobial use. Secondary infection was defined as isolation of bacterial, viral, or fungal pathogens from clinical samples at any time after the first 48 hours of admission. Isolation of *Candida* spp in respiratory samples was considered colonization for this study. Single sets of blood cultures positive for coagulase-negative *Staphylococcus* spp, *Bacillus* spp, or *Corynebacterium* spp were considered contaminants. We further described the frequency of antimicrobial administration within 48 hours of admission and during hospitalization, and we examined the risk factors associated with the prescription of antimicrobials.

### Data collection

We utilized the NYULH COVID-19 De-identified Clinical Database (CDCD) to obtain data on our study population. This database contains a data set of clinical variables extracted from electronic medical records starting January 1, 2020. Unique identifiers were stripped, and time stamps were shifted by a fixed time interval to preserve patient anonymity. The use of the CDCD is exempt from review by the NYULH’s Institutional Review Board. We used the nadir of hospital admissions between the first and second peaks of hospital admissions in the data set to delineate the first wave and later pandemic period of observation, which corresponded to July 15, 2020. The following clinical variables were extracted: patient demographics, including age, sex, pulmonary comorbidities, and smoking history, and use of any immunosuppressive medications. Clinical characteristics including oxygen requirements within 48 hours of hospital admission, peak oxygen requirements during hospitalization, WHO ordinal scale scores, hospital length of stay, ventilator days, central-line placement and overall mortality were extracted to reflect baseline characteristics of the population. We also extracted data regarding diagnostic tests performed for each patient, including blood and sputum cultures, and upper respiratory multiplex-PCR respiratory pathogen panel tests (RPP). Finally, we extracted data on administered antimicrobials, including antimicrobial classes and antimicrobial days.

### Statistical analysis

We utilized Python version 3.7 software with the open-source Impyla and Pandas libraries to execute SQL queries to fetch data from the Hadoop database via the Apache Impyla query backend. Population statistics are presented as median with interquartile range (IQR), mean with standard deviations, or proportions with 95% confidence intervals (CIs). A Kaplan-Meyer survival curve was constructed for infection-free survival up to day 28 of hospitalization, discharge, or demise. This study was approved with a waiver of informed consent by the New York University Institutional Review Board.

## Results

We identified 7,886 patients admitted to the hospital with a PCR-confirmed COVID-19 infection from January 1, 2020, to February 1, 2021. Among them, 129 patients who were aged <18 years, and 548 patients who were transferred in from other hospitals were excluded. In total, 7,209 patients were included in the final analysis. Admissions were distributed among the 3 hospitals as follows: Tisch Hospital (n = 2,617, 36.3%), NYU Brooklyn (n = 2,163, 30.0%), and NYU Long Island (n = 2,429, 33.7%). The median age of our cohort was 65 years (IQR, 55–80), and 4,202 (58.3%) were male. The most common pulmonary comorbidity was asthma (n = 953 patients, 13.2 %). Current or former history of smoking was documented in 1,778 patients (24.7%). A central venous catheter was placed in 998 patients (13.8%). Baseline characteristics of the remainder of our study population are presented in Table 1.

**Table 1.** Demographics of COVID-19 Inpatients at NYU Langone Hospitals

Variable	Grouped by Hospital			
	Overall	NYU Langone Brooklyn	NYU Langone Long Island	Tisch Hospital
Total	7,209	2,163	2,429	2,617
**Sex, no. (%)**				
Female	3,007 (41.7)	893 (41.3)	1,046 (43.1)	1,068 (40.8)
Male	4,202 (58.3)	1,270 (58.7)	1,383 (56.9)	1,549 (59.2)
Age, median y (range)	65.0 (20.0–94.0)	65.0 (20.0–94.0)	65.0 (20.0–94.0)	65.0 (20.0–94.0)
Deceased, no. (%)	1,430 (19.8)	565 (26.1)	472 (19.4)	393 (15.0)
Length of stay, median d (IQR)	5.9 (3.2–11.3)	5.2 (2.9–10.1)	6.5 (3.9–12.0)	6.0 (3.1–11.9)
**Time period, no. (%)**				
First wave	4,309 (59.8)	1,272 (58.8)	1,378 (56.7)	1,659 (63.4)
Later pandemic	2,900 (40.2)	891 (41.2)	1,051 (43.3)	958 (36.6)
**Smoking status, no. (%)**				
Current smoker	235 (3.3)	69 (3.2)	54 (2.2)	112 (4.3)
Former smoker	1,525 (21.2)	364 (16.8)	548 (22.6)	613 (23.4)
Never smoker	3,779 (52.4)	1,006 (46.5)	1,217 (50.1)	1,556 (59.5)
Unknown	1,670 (23.2)	724 (33.5)	610 (25.1)	336 (12.8)
Asthma, no. (%)	953 (13.2)	278 (12.9)	303 (12.5)	372 (14.2)
Bronchiectasis, no. (%)	127 (1.8)	31 (1.4)	31 (1.3)	65 (2.5)
Cystic fibrosis, no. (%)	2 (0.0)			2 (0.1)
COPD, no. (%)	735 (10.2)	236 (10.9)	246 (10.1)	253 (9.7)
ILD, no. (%)	239 (3.3)	77 (3.6)	75 (3.1)	87 (3.3)
**Maximum oxygen requirements <48 h of admission, no. (%)**				
Ventilator	477 (6.6)	154 (7.1)	147 (6.1)	176 (6.7)
High-flow nasal cannula	722 (10.0)	176 (8.1)	245 (10.1)	301 (11.5)
Nasal cannula	3,973 (55.1)	1,151 (53.2)	1,447 (59.6)	1,375 (52.5)
Room air	2,037 (28.3)	682 (31.5)	590 (24.3)	765 (29.2)
**Maximum oxygen requirements >48 h after admission, no. (%)**				
Ventilator	1,168 (16.2)	380 (17.6)	380 (15.6)	408 (15.6)
High-flow nasal cannula	986 (13.7)	226 (10.4)	396 (16.3)	364 (13.9)
Nasal cannula	3,604 (50.0)	1,083 (50.1)	1,247 (51.3)	1,274 (48.7)
Room air	1,451 (20.1)	474 (21.9)	406 (16.7)	571 (21.8)
**Peak WHO score in the first 48 h of admission, no. (%)**				
4.0	1,065 (25.7)	289 (24.7)	359 (24.4)	417 (27.7)
5.0	1,667 (40.2)	500 (42.8)	596 (40.5)	571 (38.0)
6.0	611 (14.7)	154 (13.2)	240 (16.3)	217 (14.4)
7.0	113 (2.7)	37 (3.2)	29 (2.0)	47 (3.1)
8.0	441 (10.6)	104 (8.9)	165 (11.2)	172 (11.4)
9.0	75 (1.8)	20 (1.7)	24 (1.6)	31 (2.1)
10.0	171 (4.1)	64 (5.5)	59 (4.0)	48 (3.2)
**Peak WHO score beyond the first 48 h of admission, no. (%)**				
4.0	1,037 (18.0)	301 (17.7)	321 (16.0)	415 (20.1)
5.0	2,582 (44.7)	754 (44.4)	905 (45.1)	923 (44.7)
6.0	738 (12.8)	145 (8.5)	312 (15.6)	281 (13.6)
7.0	42 (0.7)	15 (0.9)	15 (0.7)	12 (0.6)
8.0	207 (3.6)	52 (3.1)	56 (2.8)	99 (4.8)
9.0	170 (2.9)	30 (1.8)	57 (2.8)	83 (4.0)
10.0	995 (17.2)	401 (23.6)	340 (16.9)	254 (12.3)
Ventilator days, median (IQR)	10.0 (4.0–19.0)	6.0 (2.0–13.0)	10.0 (5.0–19.0)	14.0 (6.0–23.0)
Central line placed, no. (%)	998 (13.8)	321 (14.8)	371 (15.3)	306 (11.7)

Note. IQR, interquartile range; COPD, chronic obstructive pulmonary disease; ILI, influenza-like illness; WHO, World Health Organization.

Among all included patients, 3,379 (46.9%) had blood cultures, and 443 (6.1%) had sputum cultures performed within 48 hours of admission. In this subset, 2,037 (28.3%) of patients were on room air; 3,973 (55.1%) required nasal cannula; 722 (10.0%) required high-flow nasal cannula; and 477 (6.6%) required mechanical ventilation. Among these patients, 112 had positive blood cultures, making up 3.3% of all blood cultures sent. The most common isolate from blood was *Staphylococcus aureus*, which occurred in 20 patients (0.6%), followed by *Escherichia coli* in 14 patients (0.4%). Also, 94 patients had a positive sputum culture, comprising 21% of all sputum cultures sent at admission. The most common isolate at admission from sputum was *Staphylococcus aureus* in 31 patients (7.0%), followed by *Streptococcus pneumoniae* in 20 patients (4.5%), and *Pseudomonas aeruginosa* in 14 patients (3.2%). *Aspergillus* spp were identified in 5 patients (1.1%) among all who had a respiratory pathogen isolated <48 hours after admission. Viral coinfections were rare, with 20 (0.6%) of 3,189 RPPs sent being positive for any pathogen within 48 hours of admission. Details of RPP results are displayed in Supplementary Table 1. The most common organisms detected by cultures are summarized in Table 2.

**Table 2.** Most Frequent Organisms Isolated in Association With COVID-19 Coinfections and Secondary Infections

Organism	Coinfection (n=3,379), No. (%)^a^	Secondary Infection (n=1,565), No. (%)^a^
**Most commonly identified blood isolates**		
*Candida* spp	5 (0.1)	85 (5.4)
*Staphylococcus aureus*	20 (0.6)	33 (2.1)
*Staphylococcus epidermidis*	8 (0.2)	25 (1.6)
*Enterococcus faecalis*	9 (0.3)	23 (1.5)
*Escherichia coli*	14 (0.4)	12 (0.8)
*Enterococcus faecium*	4 (0.1)	14 (0.9)
*Klebsiella pneumoniae*	3 (0.1)	12 (0.8)
*Staphylococcus hominis*	7 (0.2)	5 (0.3)
*Pseudomonas aeruginosa*	2 (0.1)	10 (0.6)
*Serratia marcescens*	0 (0.0)	5 (0.3)
*Bacillus*	4 (0.1)	0 (0.0)
*Streptococcus gallolyticus pasteurianus*	3 (0.1)	0 (0.0)
	Coinfection (n=443)	Secondary Infection (n=816)
**Most commonly identified sputum isolates**		
*Staphylococcus aureus*	31 (7.0)	135 (16.5)
*Pseudomonas aeruginosa*	14 (3.2)	59 (7.2)
*Klebsiella pneumoniae*	3 (0.7)	51 (6.2)
*Escherichia coli*	4 (0.9)	28 (3.4)
*Klebsiella aerogenes*	1 (0.2)	30 (3.7)
*Streptococcus pneumoniae*	20 (4.5)	7 (0.9)
*Stenotrophomonas maltophilia*	3 (0.7)	24 (2.9)
*Enterobacter cloacae*	2 (0.5)	15 (1.8)
*Aspergillus* spp	5 (1.1)	12 (1.5)
*Acinetobacter calcoaceticus-baumannii*	1 (0.2)	16 (2.0)
*Serratia marcescens*	0 (0.0)	16 (2.0)
*Haemophilus influenzae*	7 (1.6)	4 (0.5)
*Legionella*	4 (0.9)	1 (0.1)
*Mycobacterium avium-intracellulare*	2 (0.5)	1 (0.1)

a Number of individuals with organism detected at each site. Percentage of patients infected with each organism over the population of all individuals tested for infection at a given site (with number of individuals tested at top of each column).

**Figure 1.**
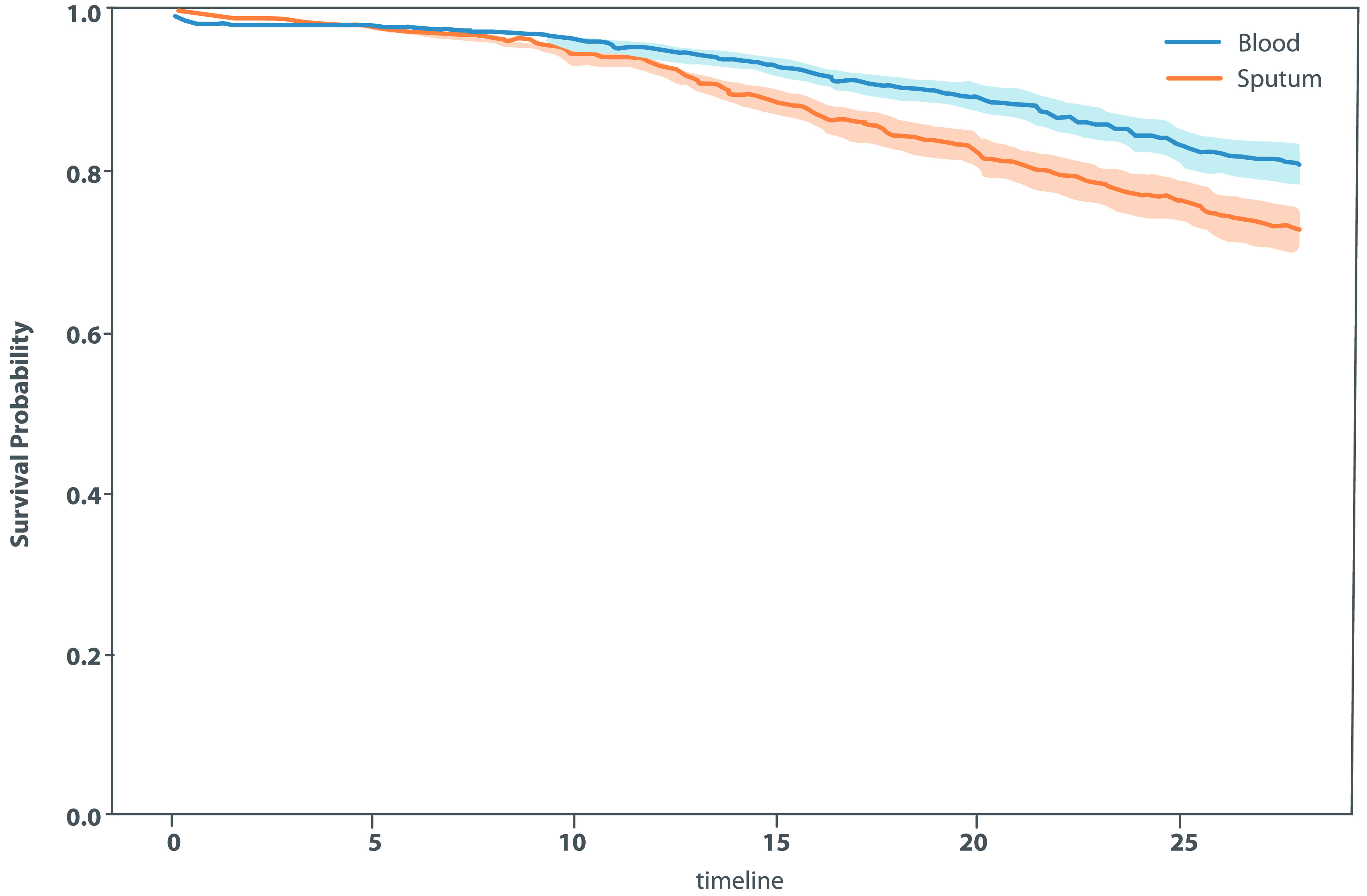
Survival Curve Of Remaining Infection-Free In Relation To The Duration Of Hospital Stay. The probability of remaining free of respiratory or bloodstream infections remains high on initial admission and decreases steadily after 10 days of hospitalization for COVID-19.

**Figure 2.**
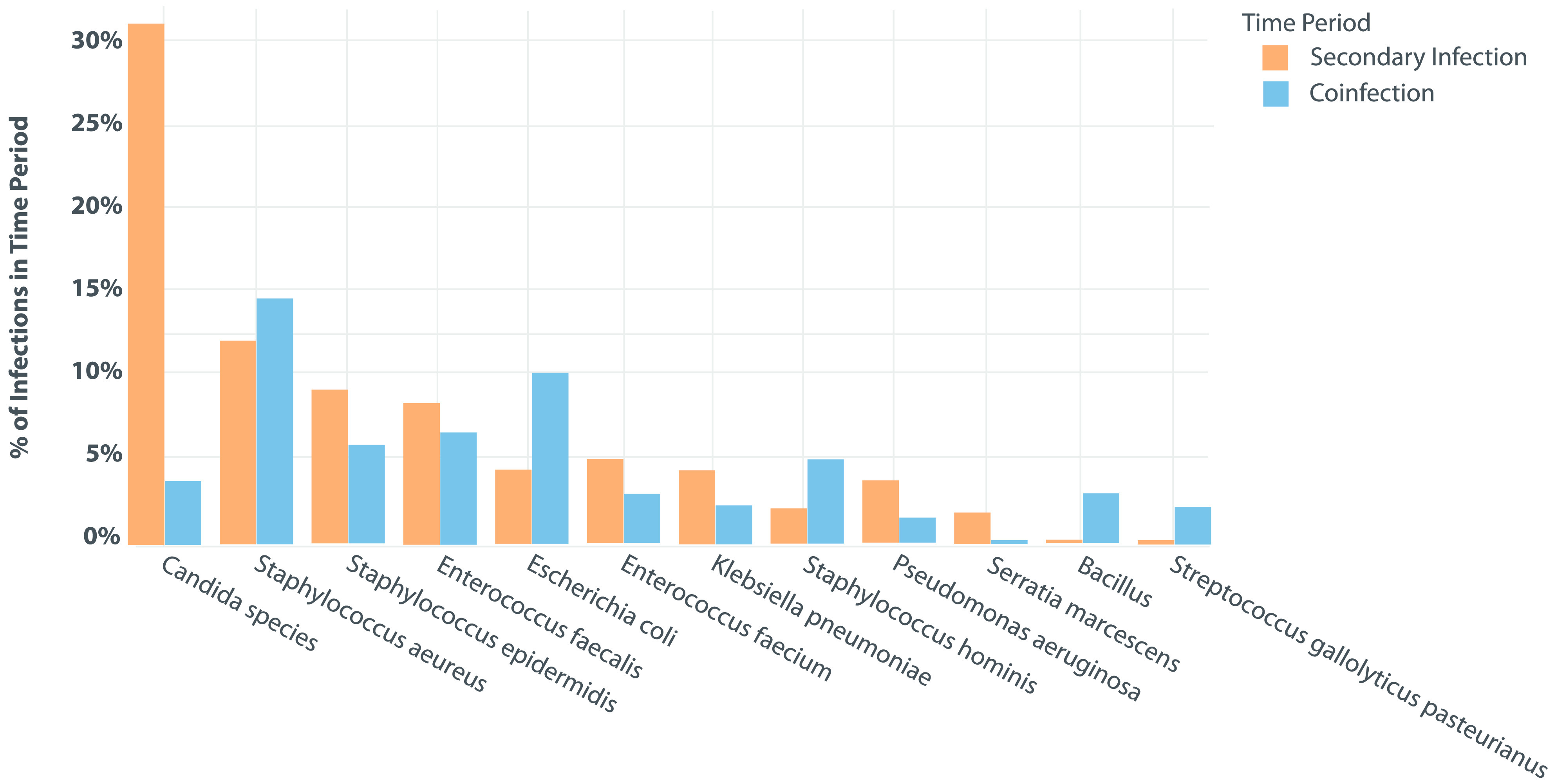
Proportion Of Organisms Isolated At Admission In Patients Admitted With COVID-19.

**Figure 3.**
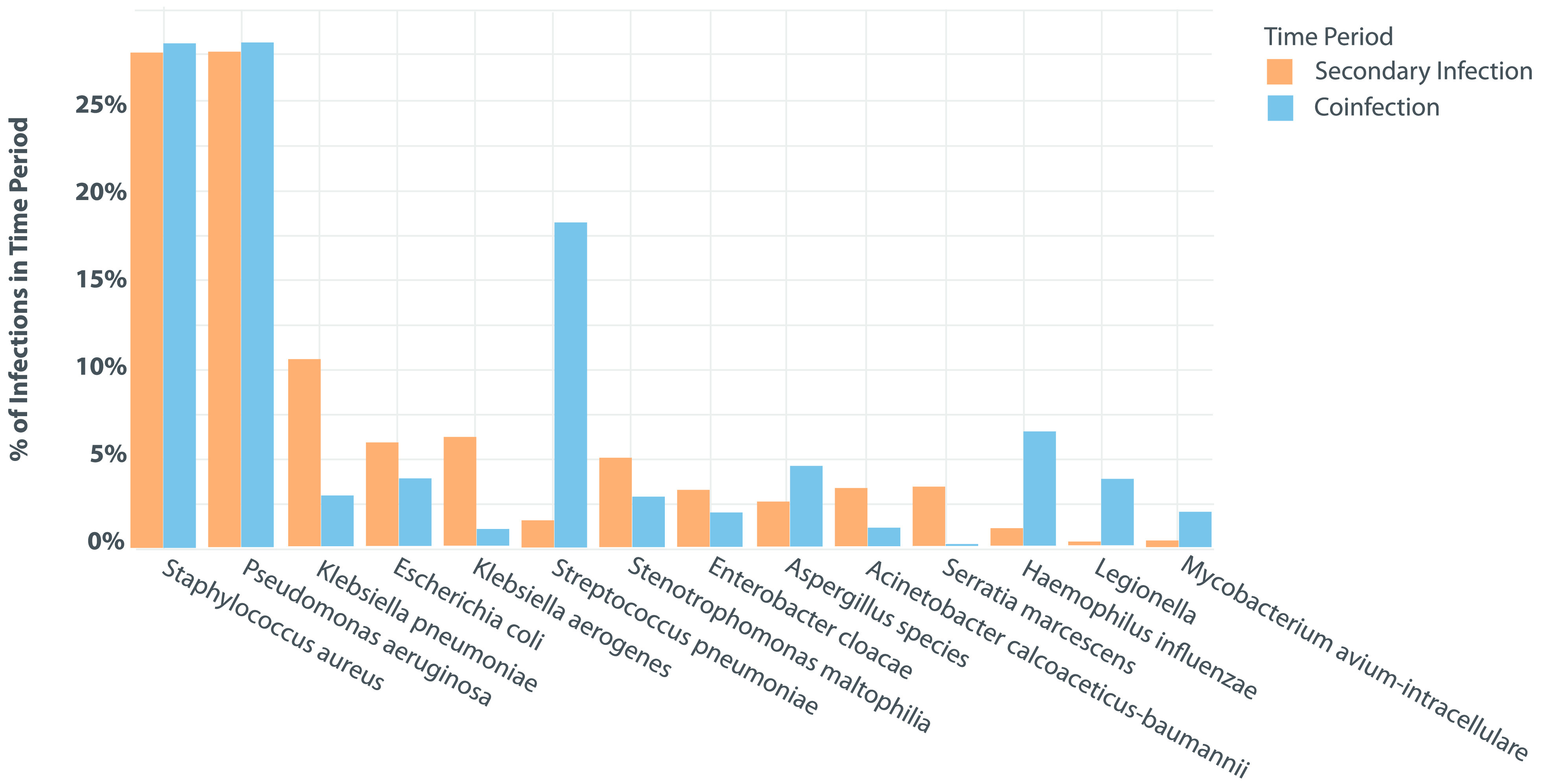
Proportion Of Organisms Isolated After 48 Hours In Patients Admitted With COVID-19.

For the remainder of the duration of admission (ie, beyond the first 48 hours), maximum oxygen requirements among the entire cohort were room air for 1,451 patients (20.1%), nasal cannula for 3,604 patients (50.0%), high-flow nasal cannula for 986 patients (13.7%), and mechanical ventilation for 1,168 patients (16.2%). Those requiring mechanical ventilation were intubated for a median of 10.0 days (IQR, 4.0–19.0). Of all included patients, 3,091 (42%) received an immunosuppressive medication, including 1,876 patients (60%) who received dexamethasone, 749 (24.2%) who received methylprednisolone, 718 (23.2%) who received tocilizumab, and 410 (13.26%) who received prednisone. In total, 222 patients (3%) had at least 1 positive blood culture, and 355 (4.9%) patients had at least 1 positive sputum culture after 48 hours of hospitalization. The most common isolates from blood were *Candida* spp, which occurred in 85 patients (5.4%), followed by *Staphylococcus aureus* in 33 patients (2.1%), *Staphylococcus epidermidis* in 25 patients (1.6%), and *Enterococcus faecalis* in 23 patients (1.5%). The most common isolates from respiratory specimens were *Staphylococcus aureus* in 135 patients (16.5%) and *Pseudomonas aeruginosa* in 59 patients (7.2%). *Aspergillus* spp were isolated from 12 patients (1.5%) with a respiratory pathogen identified >48 hours after admission. Table 2 presents the rates of coinfections and secondary infections identified in blood and sputum cultures, respectively, as well as some of the most frequently identified pathogens. Figure 1 presents the survival curve of remaining infection-free in relation to the duration of hospital stay. The probability of remaining free of respiratory or bloodstream infections remains high on initial admission and decreases steadily after day 10 of hospitalization. The proportions of organisms isolated at admission and after 48 hours of hospitalization are presented in Figures 2 and 3.

**Figure 4.**
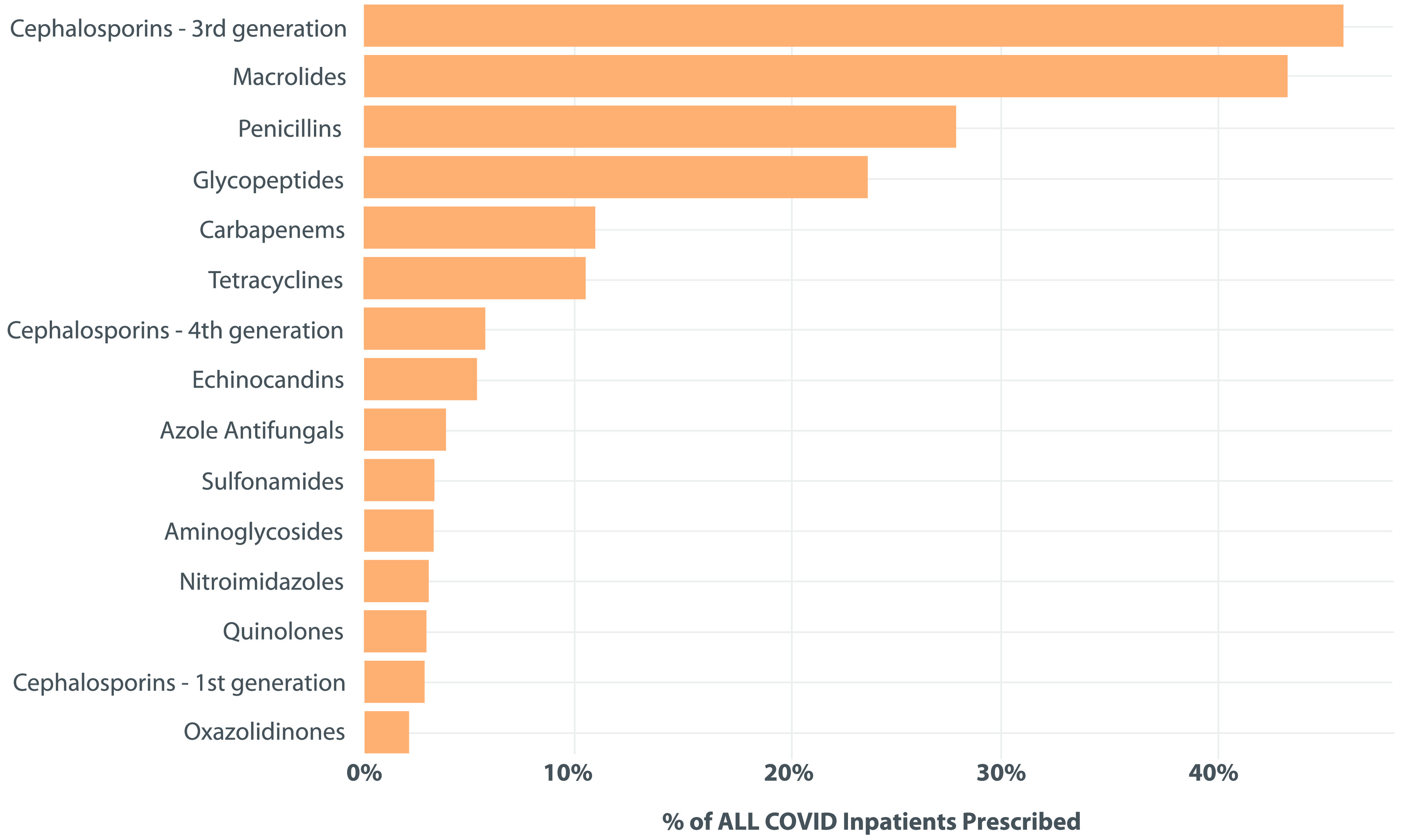
Antimicrobial Use Among Patients Admitted With COVID-19 By Pharmacologic Class. Depicted is the antibiotic use in patients admitted with COVID-19 by pharmacologic class, represented by the percentage of all COVID-19 patients who received each antimicrobial class at least once.

Antimicrobials were administered in 5,056 (70.1%) of 7,209 patients in this cohort. In total, 4,521 patients (62.7%) received a non-azithromycin antimicrobial. The most frequently used antimicrobials were third-generation cephalosporins, which were administered to 3,311 patients (45.9%). Among all study patients, 3,130 (43.4%) received azithromycin. Also, 1,183 (16.4%) received >3 classes of antibiotics, and 1,816 patients (25.2%) received antipseudomonal β-lactam antibiotics (Fig. 4). Patients treated with antimicrobials received a median of 6.0 days of therapy (Table 3a). Patients who had a copathogen identified on infective work-up received a median of 14.0 days (IQR, 7.0–29.0) of antibiotics, whereas patients who had no copathogen identified received a median of 5.0 days (IQR, 2.0–9.0) of antibiotics (Table 3b). Antimicrobial use was higher during the first wave of the pandemic; 3,549 patients (82.4%) received antimicrobials during the first wave compared to the latter period of the pandemic when only 1,507 patients (52.0%) received antimicrobials (Table 3a). Duration of hospitalization was correlated with an increasing risk of detecting a secondary pathogen (Fig. 1).

**Table 3a.** Rate of Coinfections and Antimicrobial Use in COVID-19–Positive Subpopulations

Variable	Period		
	First Wave (n=4,309), No. (%)	Later Pandemic (n=2,900), No. (%)	Overall (n=7,209), No. (%)
Blood culture sent	2,456 (57.0)	1,542 (53.2)	3,998 (55.5)
Admission blood culture sent	2,065 (47.9)	1,314 (45.3)	3,379 (46.9)
Blood culture positive	205 (4.8)	125 (4.3)	334 (4.6)
Sputum culture sent	770 (17.9)	359 (12.4)	1,129 (15.7)
Admission sputum culture sent	306 (7.1)	137 (4.7)	443 (6.1)
Sputum culture positive	307 (7.1)	133 (4.6)	449 (6.2)
Blood or sputum culture positive	439 (10.2)	224 (7.7)	663 (9.2)
Urine culture positive	293 (6.8)	272 (9.4)	565 (7.8)
Any culture positive	672 (15.6)	453 (15.6)	1,125 (15.6)
Antimicrobial received	3,549 (82.4)	1,507 (52.0)	5,056 (70.1)
Antimicrobial days, median (IQR)	6.0 (3.0–12.0)	6.0 (2.0–12.0)	6.0 (3.0–12.0)
Non-azithromycin antimicrobial received	3,054 (70.9)	1,467 (50.6)	4,521 (62.7)
>3 antimicrobial classes received	831 (19.3)	352 (12.1)	1,183 (16.4)
Antipseudomonal β-lactam received	1,183 (27.5)	633 (21.8)	1,816 (25.2)
Antimicrobials for resistant GNR received	55 (1.3)	23 (0.8)	78 (1.1)

Note. IQR, interquartile range; GNR, gram-negative rod.

**Table 3b.** Overall Antimicrobial Use in COVID-19–Positive Patients Among Those With and Without Identified Coinfections

Variable	No Coinfections Identified (n=6,084)	Coinfections Identified (n=1,125)
Antimicrobial received, no. (%)	3,970 (65.3)	1,086 (96.5)
Antimicrobial days, median (IQR)	5.0 (2.0–9.0)	14.0 (7.0–29.0)
Non-azithromycin antimicrobial received, no. (%)	3,445 (56.6)	1,076 (95.6%)
>3 antimicrobial classes received, no. (%)	613 (10.1)	570 (50.7)
Antipseudomonal β-lactam received, no. (%)	1,126 (18.5)	690 (61.3)
Antimicrobials for resistant GNR received, no. (%)	25 (0.4)	53 (4.7)

Note. IQR, interquartile range; GNR, gram-negative rod.

## Discussion

This study describes the rates of bacterial, fungal, and viral coinfections among patients admitted to the hospital with COVID-19 pneumonia, a group with high resource utilization in terms of diagnostic testing and antibiotic use. The rate of coinfections was low overall. Only 2.9% of patients hospitalized for the first time with COVID-19 were coinfected with a bacterial or fungal pathogen at admission, and only 7.9% of patients had a positive blood or sputum culture after 48 hours of admission indicative of secondary nosocomial infections. We detected 20 non–SARS-CoV-2 respiratory viral coinfections among 3,186 patients tested, none of which would meaningfully change clinical management in this population (Supplementary Table 1). Notably, detection of secondary pathogens was low during the initial week of admission (Fig. 1). Nevertheless, antimicrobial use was widespread because the clinical features of initial infection and the later inflammatory phase in COVID-19 are often challenging to differentiate from bacterial sepsis.^14^ A reduction in antimicrobial use occurred during the later stage of the pandemic, potentially due to lower case loads, better healthcare system capacity, intensified antimicrobial stewardship efforts, and improved clinician gestalt with the experience of the first wave. This pattern has also been observed in recent data from the United Kingdom and may represent the opportunity to identify targets for antimicrobial stewardship interventions.^15^

Our study had several limitations. This study was a retrospective analysis, and not all patients had a fully standardized work-up for pneumonia at admission. Sputum cultures obtained after administering antibiotics may have a lower yield of detection of bacterial infections, leading to undercounting of bacterial pathogens. It can also be challenging to determine the true clinical impact of a cultured pathogen because pneumonia caused by SARS-CoV-2 can account for significant clinical deterioration similar to sepsis from bacterial pneumonia. Only the first encounters for hospitalization for each patient were included in this analysis, which may not reflect the incidence of coinfections in readmitted patients due to secondary infections after COVID-19. We were unable to accurately describe the duration or the degree of immunosuppression or to stratify corticosteroid doses by high dose versus low dose. Outcomes of death, discharge, and long-term disability in those with coinfections or secondary infections were not reported directly in this study because reporting attributable outcomes by chart review was beyond the scope of the current de-identified data set. The risk of secondary bacterial infections after systemic immunosuppression in COVID-19 requires further investigation.

In conclusion, a low rate of coinfection occurred among patients hospitalized with COVID-19 pneumonia, and secondary infections increased with the duration of hospitalization and the use of immunomodulators. Overall, antimicrobial utilization was high, with a higher rate of utilization in the first wave of the pandemic compared to the later pandemic. With the COVID-19 pandemic continuing to significantly affect various parts of the world, resource utilization and overuse of antimicrobials warrant continued focus. Our data, obtained through the period of peak COVID-19 activity in New York City, may be used to guide the process of diagnostic decision making, optimizing resource utilization, and antimicrobial stewardship.

## Supplementary material

For supplementary material accompanying this paper visit https://doi.org/10.1017/ash.2022.51. click here to view supplementary material
