# Supplementary material for: Coinfections and antimicrobial use in patients hospitalized with coronavirus disease 2019 (COVID-19) across a single healthcare system in New York City: A retrospective cohort study
Source: Antimicrob Steward Healthc Epidemiol. 2022 May 10;2(1):e78. doi: 10.1017/ash.2022.51 (PMC9726479; doi:10.1017/ash.2022.51)
Supplement: Supplementary file 1 [file S2732494X22000511sup001.docx]

## *Supplemental table 1: Non-SARS-CoV-2 Respiratory viruses isolated in upper respiratory multiplex PCR swabs in the first 48 hours of admission*

| **Pathogen** | **Number of positive tests**  **(Total screened = 3189)** |
| --- | --- |
| **Rhinovirus/ Enterovirus** | **8** |
| **Coronavirus HKU1** | **3** |
| **Adenovirus** | **2** |
| **Human metapneumovirus** | **2** |
| **Parainfluenza type 3** | **2** |
| **Coronavirus NL63** | **1** |
| **Influenza B** | **1** |
| **Coronavirus OC43** | **1** |
| **TOTAL** | **20** |

Supplemental Table 2a: Unadjusted association of detection of co-pathogens in blood with immunomodulator therapy

| **Positive Blood Cultures Among COVID+ Inpatients Receiving Immunomodulators** | | | | | | | |
| --- | --- | --- | --- | --- | --- | --- | --- |
|  | **Isolated Before Immunomodulator** | **Isolated After Immunomodulator** | **1-Tail Z-Test** | **RR** | **RR CI** | **RR p-value** | **RR significant** |
| **Candida species** | 10 | 69 | p<0.001 | 1.38 | (1.18, 1.62) | p<0.001 | *** |
| **Staphylococcus aureus** | 14 | 23 | p=0.869 | 0.87 | (0.65, 1.18) | p=0.311 |  |
| **Staphylococcus epidermidis** | 7 | 16 | p=0.521 | 0.99 | (0.72, 1.37) | p=0.959 |  |
| **Enterococcus faecalis** | 4 | 16 | p=0.156 | 1.16 | (0.88, 1.51) | p=0.227 |  |
| **Escherichia coli** | 5 | 9 | p=0.685 | 0.91 | (0.58, 1.44) | p=0.658 |  |
| **Enterococcus faecium** | 4 | 9 | p=0.526 | 0.99 | (0.65, 1.51) | p=0.949 |  |
| **Klebsiella pneumoniae** | 2 | 8 | p=0.242 | 1.15 | (0.80, 1.66) | p=0.397 |  |
| **Pseudomonas aeruginosa** | 2 | 7 | p=0.303 | 1.11 | (0.74, 1.68) | p=0.552 |  |
| **Staphylococcus hominis** | 4 | 3 | p=0.944 | 0.61 | (0.23, 1.62) | p=0.253 |  |
| **Enterococcus** | 1 | 4 | p=0.312 | 1.15 | (0.69, 1.91) | p=0.550 |  |

Supplemental Table 2b: Unadjusted association of detection of co-pathogens in sputum with immunomodulator therapy

| **Positive Sputum Cultures Among COVID+ Inpatients Receiving Immunomodulators** | | | | | | | |
| --- | --- | --- | --- | --- | --- | --- | --- |
|  | **Isolated Before Immunomodulator** | **Isolated After Immunomodulator** | **1-Tail Z-Test** | **RR** | **RR CI** | **RR p-value** | **RR significant** |
| **Staphylococcus aureus** | 37 | 90 | p=0.683 | 0.97 | (0.84, 1.12) | p=0.640 |  |
| **Pseudomonas aeruginosa** | 13 | 46 | p=0.155 | 1.09 | (0.92, 1.29) | p=0.267 |  |
| **Klebsiella pneumoniae** | 15 | 29 | p=0.847 | 0.90 | (0.70, 1.16) | p=0.353 |  |
| **Escherichia coli** | 5 | 21 | p=0.165 | 1.12 | (0.90, 1.41) | p=0.250 |  |
| **Klebsiella aerogenes** | 8 | 16 | p=0.743 | 0.92 | (0.66, 1.27) | p=0.551 |  |
| **Stenotrophomonas maltophilia** | 2 | 19 | p=0.029 | 1.26 | (1.06, 1.50) | p=0.002 | ** |
| **Aspergillus species** | 6 | 9 | p=0.864 | 0.82 | (0.51, 1.33) | p=0.360 |  |
| **Streptococcus pneumoniae** | 8 | 6 | p=0.994 | 0.58 | (0.29, 1.17) | p=0.082 |  |
| **Enterobacter cloacae** | 1 | 12 | p=0.052 | 1.28 | (1.06, 1.56) | p=0.003 | ** |
| **Serratia marcescens** | 5 | 7 | p=0.867 | 0.80 | (0.46, 1.39) | p=0.366 |  |
